# Evaluating AlphaFold Tools and Related Scoring Functions for Protein–peptide Complex Prediction

**DOI:** 10.1093/gpbjnl/qzag026

**Published:** 2026-03-26

**Authors:** Negin Manshour, Jarett Zida Ren, Farzaneh Esmaili, Erik Bergstrom, Dong Xu (许东)

**Affiliations:** Department of Electrical Engineering and Computer Science, Christopher S. Bond Life Sciences Center, NextGen Precision Health, University of Missouri, Columbia, MO 65211, USA; Department of Electrical Engineering and Computer Science, Christopher S. Bond Life Sciences Center, NextGen Precision Health, University of Missouri, Columbia, MO 65211, USA; Department of Chemical and Biomolecular Engineering, Johns Hopkins University, Baltimore, MD 21218, USA; Department of Electrical Engineering and Computer Science, Christopher S. Bond Life Sciences Center, NextGen Precision Health, University of Missouri, Columbia, MO 65211, USA; Department of Electrical Engineering and Computer Science, Christopher S. Bond Life Sciences Center, NextGen Precision Health, University of Missouri, Columbia, MO 65211, USA; Department of Electrical Engineering and Computer Science, Christopher S. Bond Life Sciences Center, NextGen Precision Health, University of Missouri, Columbia, MO 65211, USA

**Keywords:** AlphaFold, ColabFold, Protein–peptide complex structure, Protein structure prediction assessment, Protein binding scoring function

## Abstract

Determining the three-dimensional structures of protein–peptide complexes is crucial for elucidating biological processes and designing peptide-based drugs. Protein–peptide docking has become essential for predicting complex structures. AlphaFold-Multimer, ColabFold, and AlphaFold3 are groundbreaking tools that enhance protein–peptide docking accuracy. In this study, we evaluated these three tools for predicting protein–peptide complex structures in both template-based (TB) and template-free (TF) modes. AlphaFold-Multimer excels in TB predictions and performs moderately in TF scenarios in the prediction pool; however, TF outperforms TB in the first-ranked models. ColabFold demonstrates versatility in both TB and TF settings. AlphaFold3 generates high-quality structures for more proteins, but its medium-accuracy predictions are not as good as those of AlphaFold-Multimer using a large model pool. We also assessed the performance of various scoring functions in ranking predicted protein–peptide complex structures. While the scoring function built in AlphaFold demonstrates the best performance, some other scoring functions, *e.g.*, FoldX-Stability and HADDOCK-mdscore, provide complementary information for model ranking. These findings suggest the potential for enhancing scoring functions targeting AlphaFold-based predictions by combining multiple scoring functions or using a consensus approach from many prediction models.

## Graphical abstract

**Figure:**
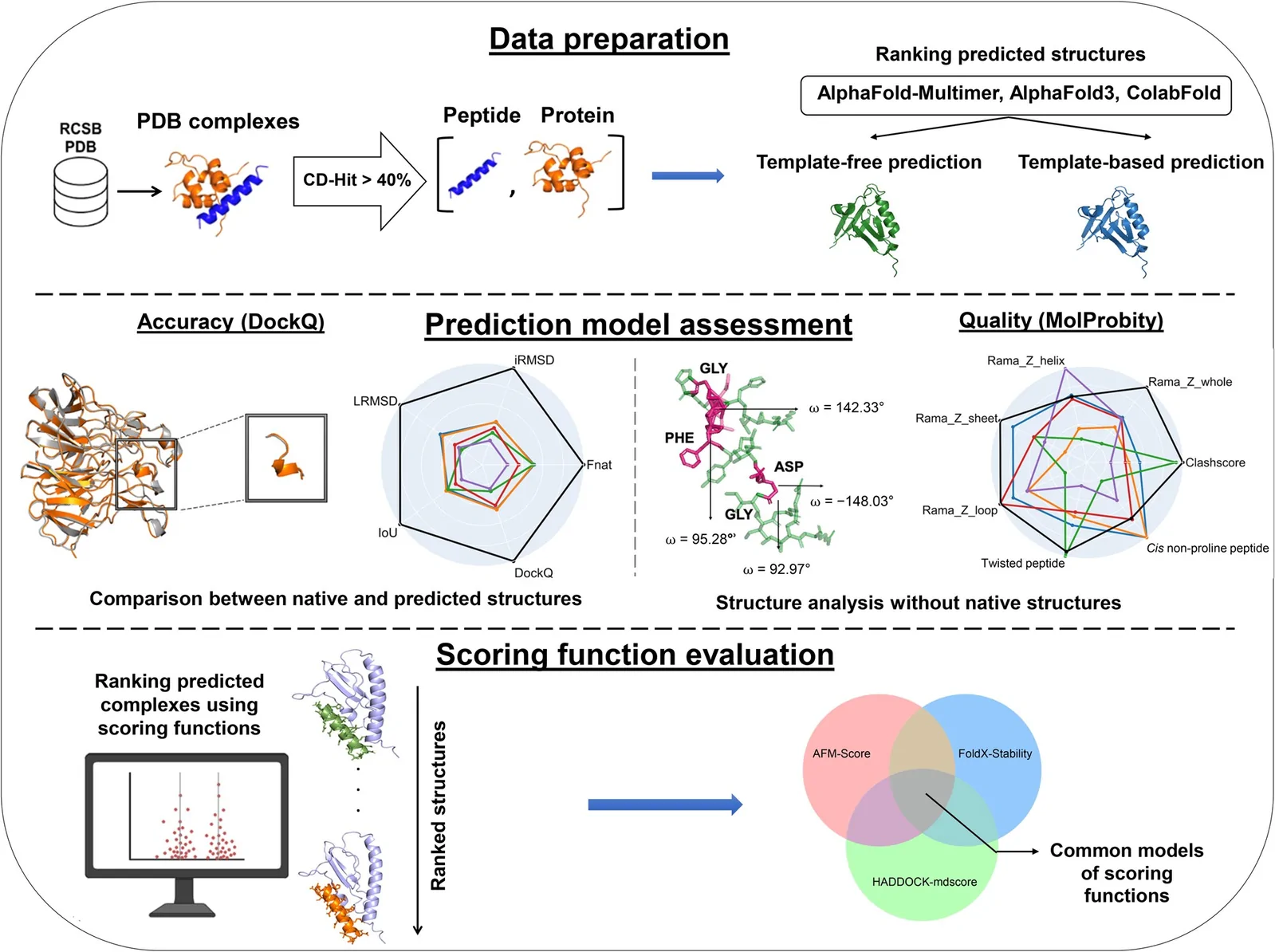

## Introduction

Protein–peptide interactions have been recognized to play crucial roles in a wide range of biological processes. The participating peptides typically consist of fewer than 50 residues. Approximately 15%–40% of protein–protein interactions are estimated to involve protein–peptide complexes [1]. Consequently, peptides have increasingly attracted attention as promising binding candidates for drug design, owing to their biochemical properties and relatively low toxicity [2]. Acquiring three-dimensional (3D) structural data of protein–peptide complexes is crucial for understanding their functional roles and malfunctions associated with diseases [3–6]. This understanding is essential for elucidating molecular mechanisms underlying protein–peptide recognition and advancing the development of peptide-based therapeutics [7,8].

To experimentally determine 3D structures of protein–peptide complexes, common techniques include cryo-electron microscopy and X-ray crystallography. While effective, these experimental methods often require extensive labor and costs [9]. Additionally, the experimental characterization of protein–peptide complex structures is considerably hindered by the dynamic and transient nature of some interactions [10]. Due to these challenges, computational methods, such as protein–peptide docking, offer valuable information for protein–peptide complex structures. Docking algorithms fall into two main categories: template-based (TB) and template-free (TF) [1,11]. In TB docking, known complex structures are used as templates to predict interactions between proteins and peptides, which is effective when similar complex structures are accessible. In contrast, TF docking, also referred to as *ab initio* docking, predicts interactions based solely on the physical and chemical properties of the molecules [12].

Previously, the prediction of protein complex structures was primarily achieved through geometry docking [13]. However, the advent of artificial intelligence (AI), particularly through applications like AlphaFold-Multimer (AFM; AlphaFold v2.3.1) [14], has recently revolutionized the field by cofolding protein–protein and protein–peptide complexes [15]. ColabFold (CF) simplifies the use of AlphaFold and AFM with Google Colab, and it predicts five TB or TF models for each protein complex. CF greatly improves the speed and ease of predicting protein complexes, thus facilitating fast advancements in structural biology [16]. AlphaFold v3.0.1 (AF3), the latest version of AlphaFold, is available through its dedicated website, providing up to five models per prediction and representing the newest version available to researchers. A locally installed version (AF3-L) was also employed, capable of generating a high number of models in both TB and TF modes [17]. Numerous studies have assessed the performance of AlphaFold; however, they have concentrated on single proteins or protein–protein complexes [18,19] (including antibody–antigen structures [20,21]), rather than on protein–peptide complexes. In this study, we focused on assessing the predicted structures of protein–peptide complexes by AFM, CF, and AF3.

A closely related research topic to model prediction accuracy is the precise identification of near-native protein–peptide interaction conformations from a large set of generated models, a process commonly known as scoring [22]. Several methods have been introduced to rank protein complex structures [23]. The evolution of scoring functions in protein structure prediction has shifted from physics-based methods, exemplified by HADDOCK [24], and empirical methods, such as AutoDock Vina [25], to knowledge-based potentials, such as FoldX [26]. Physics-based scoring functions began with force-field methods based on fundamental physical principles, incorporating solvent effects and charge features, but these methods are computationally intensive [27]. Empirical methods use simplified models of molecular interactions derived from experimental data, resulting in faster computations [28]. Knowledge-based functions use statistical potentials derived from known structural data, balancing accuracy and speed [29].

Scoring functions have further advanced with the adoption of deep learning (DL) strategies, including DOVE [30], GNN_DOVE [31], DeepRank [32], and graph neural network (GNN)-based methods (*e.g.*, DeepRank-GNN [33], DeepRank-GNN-esm [34], and InterPepRank [35]). DL-based scoring functions have been reviewed and assessed in various studies, such as those involving antibody–antigen and enzyme–inhibitor interactions [29]. Other studies have offered a comprehensive comparison of DL-based scoring functions for virtual screening [36] and ranking of the predicted protein–ligand complexes [37]. While numerous scoring functions for protein complex structure assessment have been developed, their performance depends on the methods used to predict protein–peptide complex structures, and systematic evaluation focused on the AlphaFold family is notably lacking. This gap necessitates a thorough evaluation of leading computational tools like AFM and CF, especially in the context of protein–peptide structure predictions using multiple quality and scoring functions. A comparative analysis of scoring functions will illuminate their relative strengths and pinpoint areas for potential enhancement in the methods applied.

In this study, to systematically evaluate the performance of prediction tools and scoring functions, we compiled a dataset of 60 native protein–peptide structures from the Protein Data Bank (PDB) [38,39]. Using both TB and TF modes, we employed AFM and AF3-L to predict 1000 protein–peptide structures and CF to predict 5 complex structures. Additionally, we used the AF3 web server to predict 5 models. This dataset served as a reference for assessing the effectiveness of these three prediction tools and their scoring functions.

## Results

We conducted a comprehensive two-part evaluation and analysis of protein–peptide complex structures (**Figure 1**). The first part focused on assessing the quality of predicted structures, while the second part aimed to analyze various scoring functions. To this end, we employed two quality measures, DockQ [40] and MolProbity [41,42], as well as four distinct scoring functions. The insights derived from this study provide a nuanced understanding of the accuracy, capabilities, and limitations of AFM, CF, AF3, and AF3-L, as well as the efficacy of each scoring function employed.

**Figure 1:**
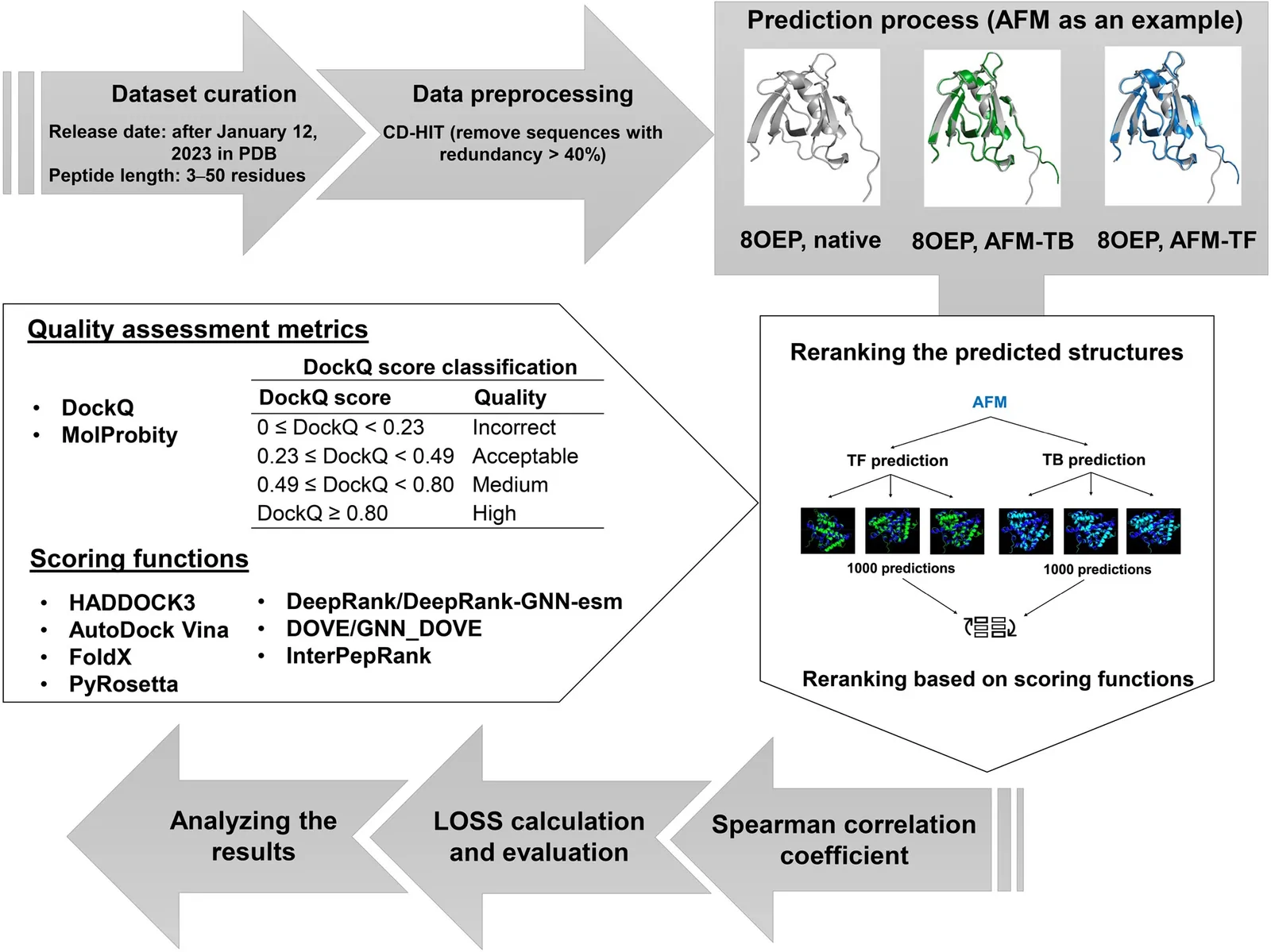
Overview of the study workflow The dataset comprised protein–peptide complexes deposited in the PDB after January 12, 2023, with standard amino acids in peptides of 3–50 residues and no overlap with AlphaFold’s training dataset. All sequences containing nonstandard residues were removed. The dataset was further refined using CD-HIT to eliminate sequences with > 40% redundancy, resulting in 60 diverse protein–peptide complexes for the evaluation pipeline. All structures of sample sequences were predicted by AFM, CF, and AF3/AF3-L. The models generated by AFM, CF, and AF3-L were categorized into TB and TF based on the prediction mode. These models were subsequently evaluated using various quality assessment metrics and scoring functions. The Spearman correlation coefficient (*ρ*) was calculated for the rankings produced by all scoring functions compared to the ranking by DockQ. A loss parameter was defined to evaluate the accuracy of each scoring function in identifying the most near-native structure from the pool. PDB, Protein Data Bank; CD-HIT, Cluster Database at High Identity with Tolerance; AFM, AlphaFold-Multimer; CF, ColabFold; AF3, AlphaFold3 web server; AF3-L, local AlphaFold3; TB, template-based; TF, template-free.

### Quality analysis

In this section, we provided a detailed analysis using DockQ to assess the prediction accuracies of three leading protein structure prediction tools, AFM, CF, and AF3/AF3-L, across 60 samples against our ground-truth (GT) dataset. Additionally, we used MolProbity to diagnose structural problems in the predicted models. For each protein, AFM and AF3-L produced 1000 predictions, while CF and AF3 produced 5. We compared the predicted complex structural models with their corresponding native structures using DockQ. We segmented the datasets into four categories based on DockQ prediction quality: high, medium, acceptable, and incorrect. This classification evaluates the practicality of the predicted structures for subsequent biological and biochemical analyses. We used MolProbity to assess the quality of complex structures in terms of their potential issues without knowing the native structures.

Our analysis acknowledges the inherent trade-offs between sampling depth and computational efficiency across these methods. While the extensive sampling of AF3-L and AFM enables broader conformational exploration and improved scoring reliability, the limited sampling of CF and AF3 substantially reduces computational demands and makes them more accessible to a wider range of users. In particular, the AF3 web server generates only the top 5 models, but it is widely used. The locally installed AF3-L was employed exclusively for the 1000-model comparison with AFM to ensure equal sampling in large-pool evaluations. This multi-level analytical approach allows us to evaluate both the prediction quality of the best-selected models under realistic user conditions and the benefits of extensive sampling relative to computational efficiency.

#### DockQ

DockQ is a scoring tool used to evaluate the quality of protein–protein docking models by comparing predicted structures with reference (native) structures. It provides a single metric that combines interface root-mean-square deviation (iRMSD), ligand RMSD (LRMSD), and fraction of native contacts (Fnat), facilitating the assessment of docking accuracy and model quality [40,43]. DockQ scores, ranging from 0 to 1, assess the accuracy of predicted models against native PDB structures. Scores above 0.23 are considered acceptable by the CAPRI standards [44] (Figure 1). These scores indicate the quality of the interaction interface between the protein and the peptide relative to the native structure. **Figure 2** illustrates the evaluation results for TB and TF modes in large model pools for AFM and AF3-L and assesses the quality of top-ranked models from AFM, CF, and AF3. The AFM first-ranked and top-five selections were unchanged when sampling increased, justifying a direct comparison between AF3 and CF.

**Figure 2:**
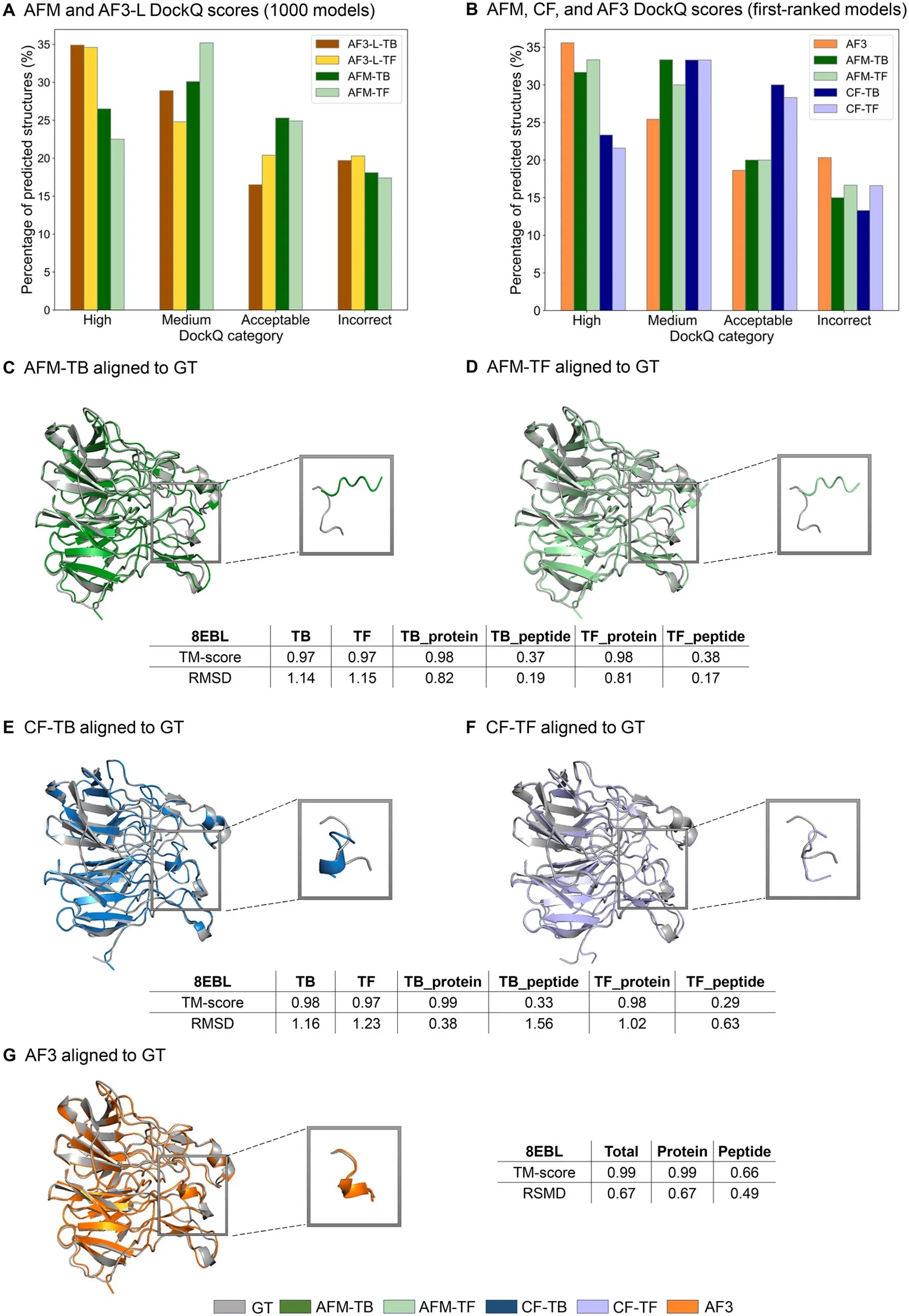
Evaluation of protein–peptide complex structures predicted by AFM, CF, and AF3/AF3-L **A**. Percentage of structures predicted by AFM and AF3-L across 1000 models in different categories of DockQ. **B**. Percentage of first-ranked structures predicted by AFM, CF, and AF3 in different categories of DockQ. **C**.–**F**. Comparative analysis of protein–peptide complex predictions for sample 8EBL by AFM in TB mode (C), AFM in TF mode (D), CF in TB mode (E), and CF in TF mode (F), respectively, assessed against the GT structure using TM-score and RMSD. **G**. Comparative analysis of structure predicted by AF3 against the GT structure. GT, ground-truth; RMSD, root-mean-square deviation; TM-score, template modeling score.

As shown in Figure 2A, for AFM, the TF mode primarily produced structures in the medium category (35.2%), whereas the TB mode generated more high-quality predictions (26.5%) than TF (22.5%), demonstrating the critical role of template information in achieving optimal prediction accuracy. AF3-L outperformed AFM in both modes for predicting high-quality structures, with approximately 34.9% (TB) and 34.6% (TF) of predictions falling into the high category. In AF3-L, TB achieved a higher proportion of medium-quality models (28.9%) than TF (24.8%), while TF produced a slightly higher proportion in the acceptable category (20.4% in TF *vs*. 16.5% in TB). The incorrect category percentages were similar between TB and TF for AF3-L (19.7% and 20.3%, respectively). The side-by-side comparison of AFM and AF3-L reveals clear performance differences. AF3-L consistently yielded a larger fraction of high-quality structures than AFM in both TB (34.9% *vs*. 26.5%) and TF (34.6% *vs*. 22.5%) modes. AFM maintained higher proportions of medium-quality structures in both TB (30.1%) and TF (35.2%) modes than AF3-L, suggesting that AFM predictions more often fell into the medium range, while AF3-L shifted more models into the high category. These results underscore AF3-L’s advantage in producing high-quality structures, while highlighting AFM’s relative strength in generating medium-quality predictions.

In protein structure prediction, importance is often placed on the quality of the first-ranked structures selected by prediction tools without knowing the native structures, as these predictions are usually used in subsequent biological analyses and functional studies. Consequently, we compared the quality of first-ranked protein structures predicted by AFM and CF using DockQ scores in both TB and TF modes (Figure 2B). For AFM, TF slightly outperformed TB in the high category (33.3% *vs*. 31.6%). Interestingly, although TB showed better average performance than TF across all prediction models, the 1000 models predicted by TF were expected to be more diverse than those generated by TB, and AFM had a strong capacity to select the best model from the pool. A substantial consistency between TB and TF modes was demonstrated by CF, with a near-equal distribution of high- and medium-quality predictions observed, although its TB mode was slightly better. This may be because CF predicted a small number of models in both TB and TF modes, resulting in little diversity in the model pool. AF3 generated the highest proportion of high-quality structures, with 35.6% of the first-ranked models falling into this category, followed by 25.4% in the medium category.

Figure 2C–G illustrate the first-ranked protein–peptide structures predicted by AFM, CF, and AF3 for the same sample (PDB ID: 8EBL), using both TB and TF modes for AFM and CF. To analyze the prediction accuracy of each method, we calculated the template modeling score (TM-score) [45] and root-mean-square deviation (RMSD) [46] for the entire complex structure, as well as for the protein and peptide components separately. Based on the results, the accuracy of protein–peptide complex structure predictions by AFM, AF3, and CF was high, with TM-scores around 0.97–0.99 and RMSD values around 0.67–1.23. For protein predictions, the TM-scores were within the range of 0.98–0.99; however, the RMSD values varied, with the lowest observed for CF-TB and the highest recorded for CF-TF. The predicted peptide structure was of lower quality than the protein, with TM-scores decreased to 0.37, 0.38, 0.33, 0.29, and 0.66 for AFM-TB, AFM-TF, CF-TB, CF-TF, and AF3, respectively. AF3 outperformed both AFM and CF in terms of TM-score for the predicted peptide.

The distributions of all DockQ parameters, including Fnat, iRMSD, LRMSD, DockQ, and intersection over union (IoU), are shown in Figures S1–S5. While AF3 had the highest proportion of high-quality scores (DockQ ≥ 0.80), its average prediction accuracy, as indicated by the median and quartiles in Figure S4, was less favorable than that of other tools. Additionally, the radar plot in **Figure 3**A shows the median values of the DockQ parameters. This analysis underscores the superior performance of AFM-TB and AFM-TF in producing high-quality and consistent protein–peptide docking predictions.

**Figure 3:**
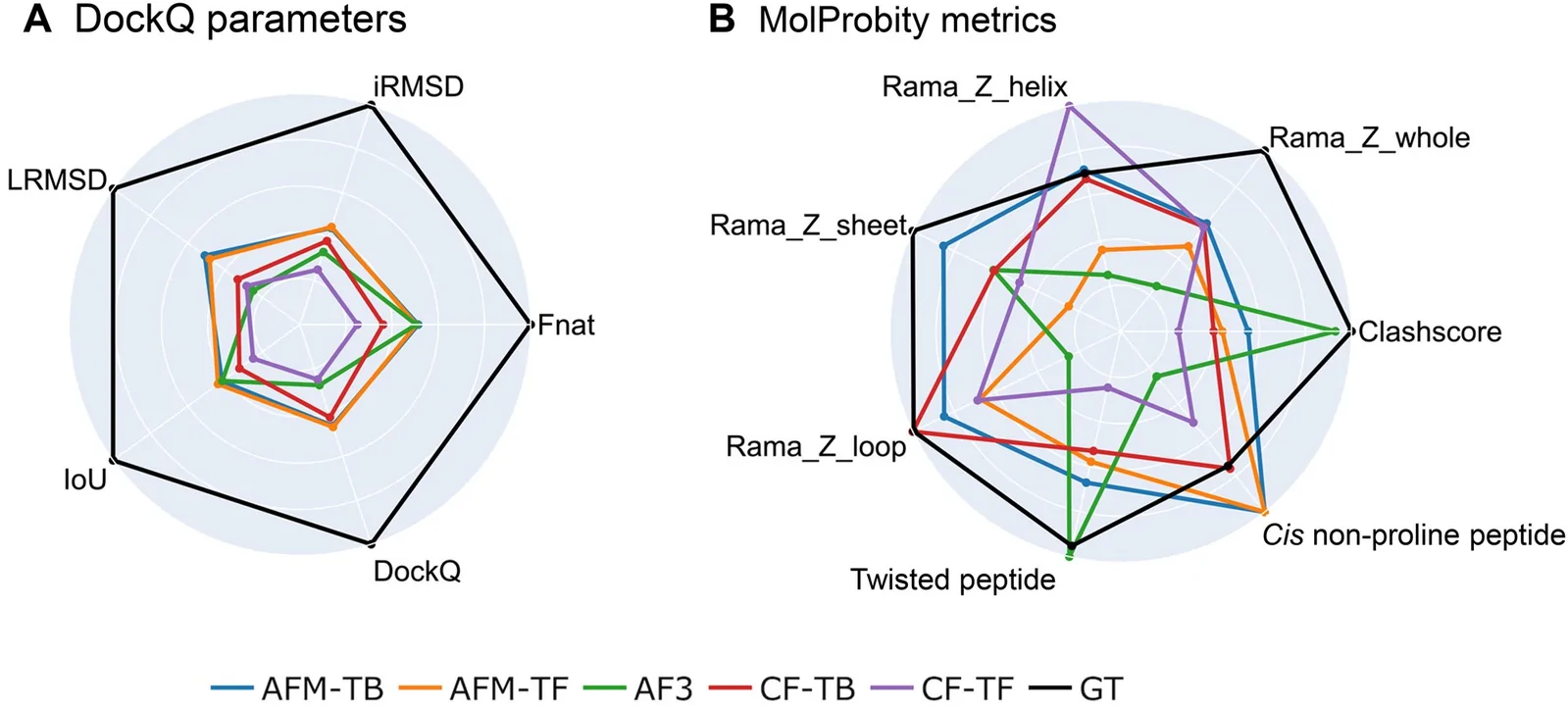
Evaluation of prediction accuracy and structure quality for the first-ranked protein–peptide models predicted by different methods For each metric in the radar plots, the best-performing method is positioned at the outermost circle, the worst at the innermost circle, and all others are scaled linearly between them. **A**. Radar plot of median values for DockQ parameters (Fnat, iRMSD, LRMSD, IoU, and DockQ) across five methods, with GT included as a reference ( score = 1 for all metrics). **B**. Radar plot of MolProbity metrics. Clashscore is represented by its median value (lower is better). Rama_Z scores (helix, sheet, loop, and whole) are represented by their RMS values (lower is better). GT shows the best overall performance, with the lowest RMS values across all Rama_Z metrics except Rama_Z_helix. Fnat, Fraction of Native Contacts; iRMSD, interface root-mean-square deviation; LRMSD, ligand root-mean-square deviation; IoU, intersection over union; RMS, root mean square.

To further investigate the effect of sampling depth on prediction accuracy, we analyzed the changes in the best DockQ score obtained as the number of generated models increased. We selected three representative protein–peptide complexes with varying initial prediction quality: 8AHS (low initial score), 8B58 (medium), and 8Q7K (high). Models were ranked by DockQ, and the highest score observed was tracked as progressively larger subsets of the model pool were considered. As shown in Figure S6, 8AHS exhibited a steep rise in DockQ score from 0.08 to ∼ 0.72 within the first 100 models, plateauing above 0.73 after ∼ 500 models. For 8B58, the score improved gradually from 0.52 to 0.67, with most gains occurring between 1000 and 1500 models. In 8Q7K, which started at 0.905, the improvement was modest but consistent, reaching ∼ 0.917 with extensive sampling. These results demonstrate that large model pools can yield substantial gains for low- and medium-quality starting points, while still providing measurable improvement for high-accuracy predictions.

Additionally, to statistically validate our comparisons between prediction methods, we performed paired *t*-tests across all five approaches. The analysis revealed statistically significant differences between all method pairs, with varying degrees of distinction. The most pronounced differences were observed between AF3 and AFM-TB (*P* = 2.107E−60), while CF variants (CF-TB *vs*. CF-TF, *P* = 2.467E−10) showed the smallest, though still highly significant differences. The AlphaFold family methods exhibited more extreme statistical differences from each other than might be expected given their architectural similarities. A complete heatmap of *P* values and methodological details are provided in Figure S7.

To further evaluate the relative performance of AlphaFold-based methods within the broader landscape of protein–peptide docking tools, we conducted a comparative analysis against established methods including GalaxyPepDock [47], MDockPeP2 [48], pepATTRACT [49], and Robetta [50] (Figure S8). This analysis was performed on 10 representative complexes selected to meet the size constraints of all tested methods. The results demonstrate that AFM, in both TB and TF modes, achieves the highest proportion of high-quality predictions (DockQ ≥ 0.8) among all methods tested. Traditional docking tools show notably lower accuracy, confirming the superior performance of AlphaFold-based methods in protein–peptide docking.

#### MolProbity

MolProbity is a popular validation tool for assessing protein structure quality through stereochemistry and atomic interactions. It examines amino acid residue conformations, angles, and bond lengths, pinpointing deviations from standard values. The MolProbity score combines the Clashscore, rotamer, and Ramachandran evaluations into a single, comprehensive score [42]. In this study, we used the MolProbity score, along with analyses of twisted peptides and *cis* non-proline conformations, to evaluate protein–peptide complex structures. As shown in **Figure 4**A, MolProbity scores provide the framework for our analysis, offering a detailed look at the quality of structural predictions from AFM, CF, and AF3. These scores inform us of the accuracy distribution for AFM-TB, AFM-TF, CF-TB, CF-TF, and AF3, with lower MolProbity scores indicating higher structural quality.

**Figure 4:**
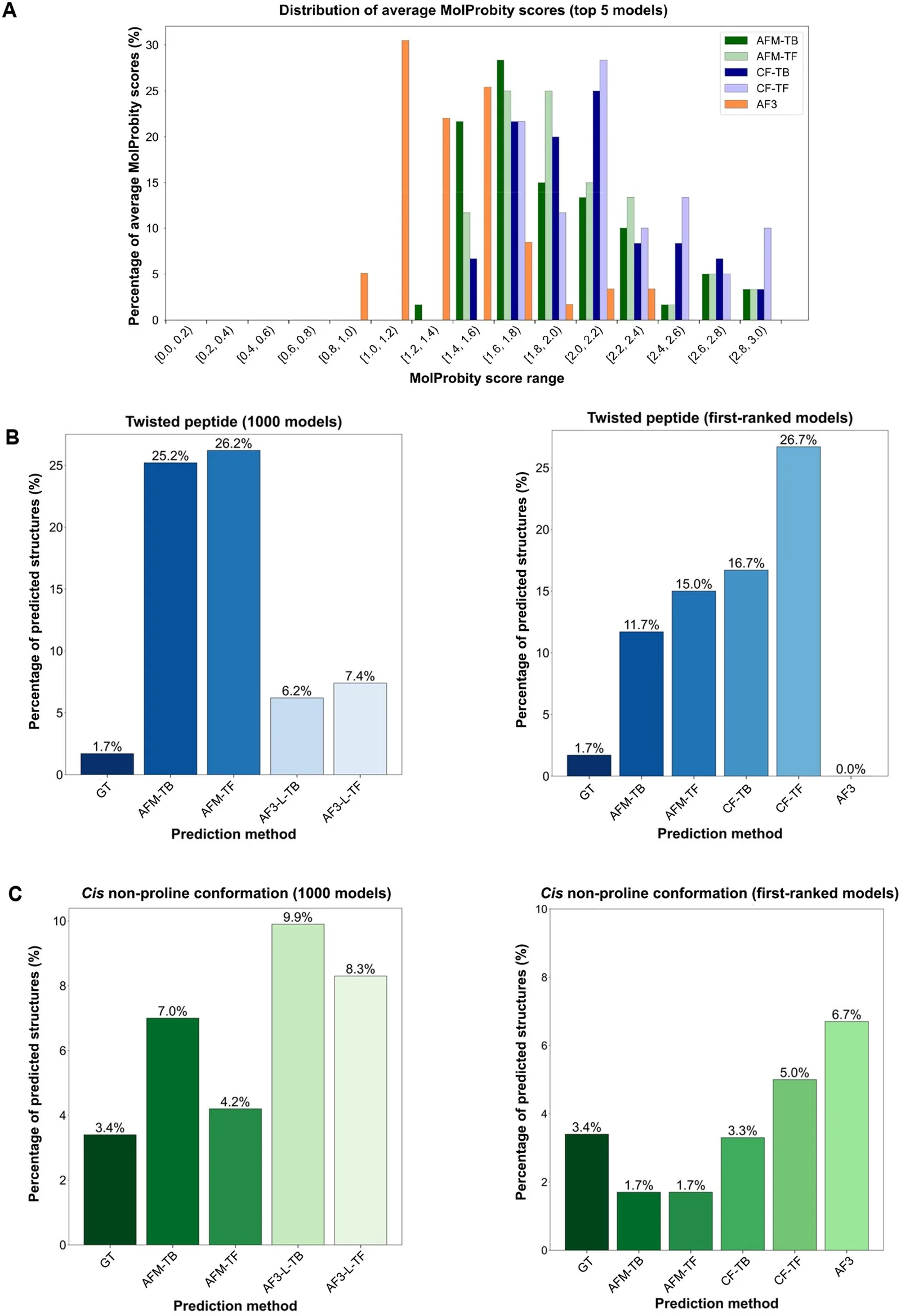
Evaluation of MolProbity scores, twisted peptides, and ***cis*** non-proline conformations **A**. Distribution of average MolProbity scores for the top 5 models predicted by AFM (TB and TF), CF (TB and TF), and AF3. Lower MolProbity scores indicate higher structural quality. **B**. Percentage of predicted structures containing twisted peptides. Left: all 1000 models generated by AFM and AF3-L. Right: first-ranked models generated by AFM, CF, and AF3. **C**. Percentage of predicted structures containing *cis* non-proline conformations. Left: all 1000 models generated by AFM and AF3-L. Right: first-ranked models generated by AFM, CF, and AF3. Predicted structures with fewer twisted peptides, fewer *cis* non-proline conformations, or those closer to the GT structures are preferred.

The AF3 model demonstrated superior stereochemical quality among the top 5 predictions, with the highest proportion (30.5%) falling within the 1.0–1.2 MolProbity score range, followed by 25.4% in the 1.4–1.6 range and 22.0% in the 1.2–1.4 range. AF3 also maintained a low representation in higher MolProbity score ranges, with less than 3.4% of models above 2.2, indicating consistently high-quality structures. In comparison, AFM-TB and AFM-TF showed broader distributions. AFM-TB peaked at 1.6–1.8 (28.3%), with notable proportions in the 1.4–1.6 (21.7%) and 1.8–2.0 (15.0%) ranges, while AFM-TF also peaked at 1.6–1.8 (26.7%), with a substantial proportion in the 1.8–2.0 (23.3%) range. Both AFM approaches had appreciable percentages in the 2.0–2.2 range (13.3% for TB and 15.0% for TF), reflecting more frequent lower-quality predictions than AF3.

The CF models exhibited even more dispersed distributions across the MolProbity score spectrum. CF-TB had major peaks in the 2.0–2.2 (23.3%) and 1.6–1.8 (21.7%) ranges, along with substantial proportions above 2.2, indicating a higher frequency of structures with lower stereochemical quality. CF-TF showed its largest proportion in the 2.0–2.2 (28.3%) range, followed by 1.6–1.8 (21.7%), and also had notable proportions above 2.2, including 2.8–3.0 (10.0%). Overall, AF3 generated the highest proportion of low MolProbity scores; AFM-TB and AFM-TF delivered intermediate performance with broader quality variation; and CF-TB and CF-TF showed the widest distribution with the poorest overall stereochemical quality.

Significant differences among these prediction methods were observed in the percentage of twisted peptides within protein–peptide complexes when evaluating all 1000 models per method (Figure 4B). Only 1.7% of the GT structures contained twisted peptides. AF3-L showed relatively low percentages, with 6.2% for TB and 7.4% for TF, indicating better preservation of correct peptide geometry than AFM. In contrast, AFM produced substantially higher percentages, with 25.2% for TB and 26.2% for TF, meaning that more than a quarter of its predictions contained twisted peptides. Twisted peptide conformations arise from excessive backbone torsion or strained dihedral angles that are rarely observed in experimentally determined structures; the lower incidence in AF3-L suggests that its modeling framework imposes stronger geometric regularization, whereas AFM appears more prone to sampling and retaining conformations with distorted peptide backbones.

For the first-ranked models, compared with the baseline of 1.7% of the GT structures containing twisted peptides, AFM-TB had 11.7%, followed by AFM-TF and CF-TB with 15.0% and 16.7%, respectively. The lowest-quality first-ranked models were produced by CF-TF, with the highest percentage at 26.7%. Interestingly, 0.0% was observed for AF3, implying that no twisted peptides were identified in its first-ranked models. We note that, except for those generated by CF-TF, first-ranked models had fewer twisted peptides than all other models on average. This indicates that AF3’s model selection strategy is highly effective at promoting geometrically plausible peptide conformations to the top rank, even when such distortions may exist in lower-ranked models. In contrast, the higher twisted-peptide percentages observed for AFM and CF suggest that their ranking procedures are less sensitive to geometric quality when selecting the first-ranked model.

We conducted a comparative analysis of the presence of *cis* non-proline peptides (Figure 4C; see Figure S9 for an example). When evaluating all 1000 models per method, 3.4% of the GT structures contained *cis* non-proline peptides. AF3-L generated higher percentages of 9.9% for TB and 8.3% for TF, while AFM produced lower percentages of 7.0% for TB and 4.2% for TF. *Cis* non-proline conformations are rare in experimentally determined structures due to steric strain and disrupted hydrogen bonding. Higher percentages observed for AF3-L indicate an occasional preference for strained backbones, while AFM, particularly in TF mode, tends to underpredict these conformations, showing a bias towards canonical *trans* geometries. These differences may influence how each method models flexible peptide backbones and thus should be considered when choosing prediction tools. For the first-ranked models, AFM-TB and AFM-TF each showed 1.7%, below the GT baseline, while CF-TB and CF-TF yielded 3.3% and 5.0%, respectively. AF3 produced the highest percentage among the first-ranked models (6.7%).

The distributions of Ramachandran scores [51] obtained from the MolProbity tool for first-ranked models are shown in Figures S10–S13, which illustrate the distributions of Rama-Z-whole scores, Rama-Z-helix scores, Rama-Z-sheet scores, and Rama-Z-loop scores, respectively. Rama-Z scores ranging from −1 to 1 indicate that the dihedral angles are very similar to those found in GT structures. Rama-Z scores in the ranges of −2 to −1 and 1 to 2 indicate that the models slightly deviate from the normal dihedral angle distributions but are still acceptable. Rama-Z scores beyond ±2 mark significant deviations from the expected values, suggesting potential issues with the protein models, such as unusual conformations that could be errors. All methods performed similarly in these Rama-Z scores, and all were significantly worse than the GT. In particular, the GT showed a significantly different distribution of Rama-Z-helix scores from other methods, with a higher proportion of models falling within the range of −1 to 1 (mostly negative values for GT *vs.* mostly positive values for other methods). This suggests that the helices generated by all models are not quite protein-like. AF3 had better Rama-Z-helix and Rama-Z-sheet scores but worse Rama-Z-loop scores than other methods. The distribution of Clashscores for first-ranked models across different protein modeling methods is shown in Figure S14. AF3 showed the lowest Clashscore among all models, close to that of the GT, while other methods had significantly higher Clashscores. As shown in Figure 3B, the radar plot of the MolProbity parameters displays the performance profile of each model for all first-ranked models across all protein–peptide samples. AF3 showed the best overall geometry for twisted peptides and the most favorable Clashscores.

**Figure 5** presents example protein–peptide structures predicted by AFM-TB, AFM-TF, and AF3, along with their MolProbity metrics. Figure 5A displays the hydrogen bonding between the protein and peptide in the 8HLO sample. It demonstrates that the hydrogen bonding in the native structure is stronger than that in the structures predicted by AFM-TB, AFM-TF, and AF3. Figure 5B depicts the *cis* non-proline conformations in the peptide portion of the 8EBL sample. As illustrated, the AFM-TB model exhibited a *cis* non-proline conformation, indicated by its ω angle (a *cis* peptide is characterized by an ω angle between −30° and +30°, whereas a *trans* peptide is defined by an ω angle greater than +150° or less than −150° [52]). In contrast, this conformation was absent in the native structure, as well as in the structures predicted by AFM-TF and AF3. Figure 5C shows the presence of twisted peptides (ω angles that deviate more than 30° from planar [52]) in the peptide portion of the 7Z7C sample for both AFM-TB and AFM-TF, while AF3 does not show any twisted peptide in that region. In this instance, the AFM-TB model exhibited more twisted peptides than the AFM-TF model, despite the benefit of using templates for structure prediction. Figure 5D depicts the clashes in the peptide portion of the 8AFI sample.

**Figure 5:**
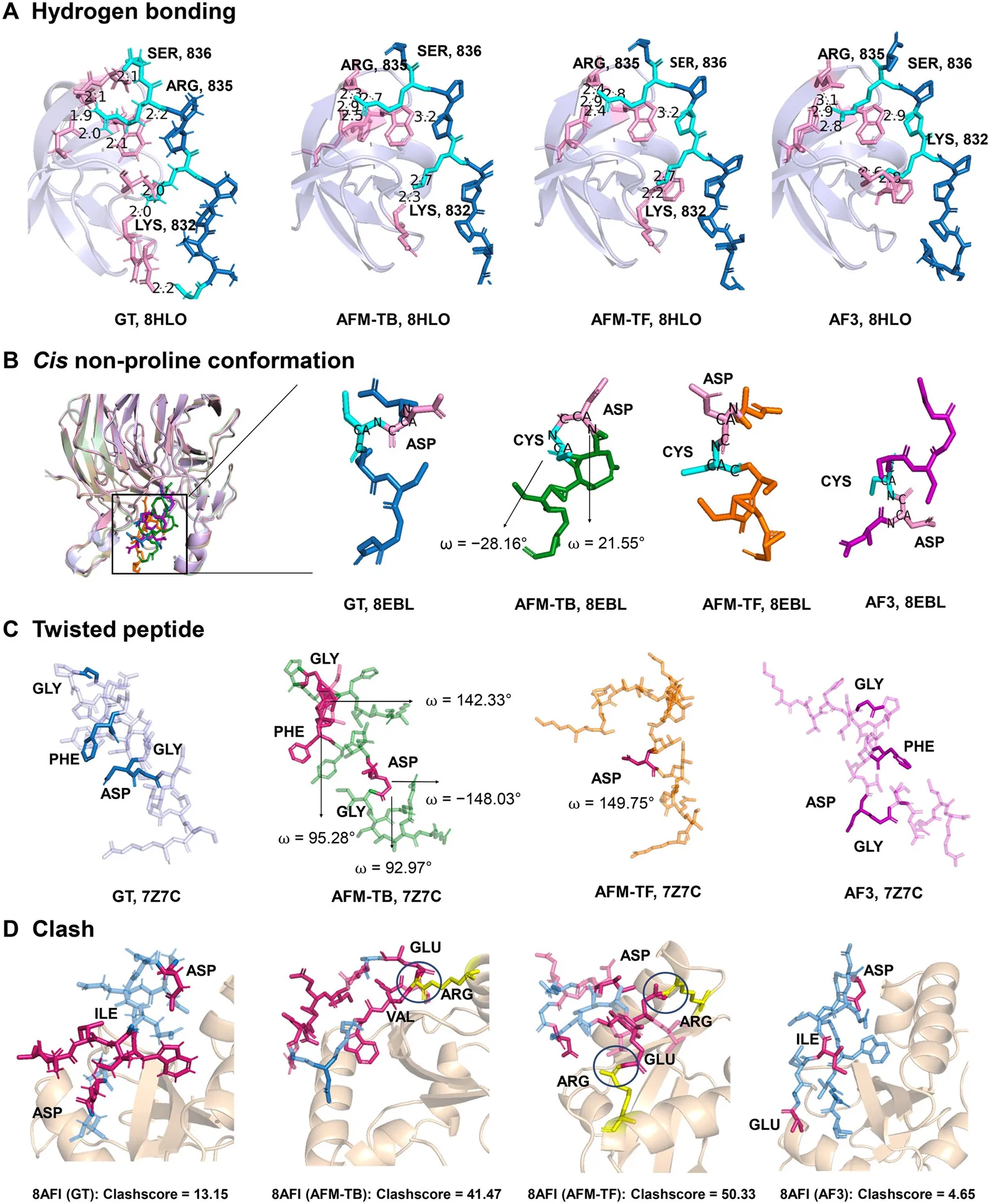
Representative examples of hydrogen bonding, ***cis*** non-proline conformations, twisted peptides, and clashes in predicted protein–peptide complex structures **A**. Hydrogen bonding across the protein–peptide interface of sample 8HLO. Hydrogen bonds are shown for the GT structure and the structures predicted by AFM-TB, AFM-TF, and AF3. **B**. *Cis* non-proline conformations in sample 8EBL. A predicted structure ranked 511 for both AFM-TB and AFM-TF is shown here, as top-ranked structures did not have any *cis* non-proline peptides. Corresponding residues across all structures are indicated in pink and cyan. **C**. Twisted peptides in the peptide region of sample 7Z7C. Twisted peptides, characterized by unusual omega (ω) dihedral angles, deviate from standard *cis* (ω ≈ 0°) or *trans* (ω ≈ 180°) conformations. Examples of such deviations include ω angles of 92.97°, 95.28°, −148.03°, and 142.33° in the AFM-TB model, and 149.75° in the AFM-TF model, none of which are close to the canonical *cis* or *trans* conformations, indicating twisted peptide bonds. **D**. Clashes at the peptide site of sample 8AFI, along with the corresponding Clashscore for each entire complex structure. The AF3 model exhibited the fewest clashes at the peptide site in sample 8AFI.

In addition, we analyzed the impact of relaxation on structural quality using a subset of 10 diverse samples by applying relaxation to the top 5 models for each sample. As shown in Figure S15A–C, relaxed AFM models demonstrated substantial improvements in stereochemical properties. The relaxation process markedly reduced the proportion of incorrect models in both TB and TF modes (to 2% in the top 5 models), with Relaxed-TB eliminating incorrect predictions among first-ranked models. MolProbity metrics revealed significant quality enhancements, with Relaxed-TB and Relaxed-TF protocols exhibiting drastically lower percentages of models with Clashscores above the threshold (1.4%–2.1%) compared to default AFM (13.8%–16.3%) and even AF3 (4.3%). These results indicate that implementing the relaxation step can significantly enhance model quality without sacrificing interface accuracy.

### Evaluation of scoring functions

In this section, we demonstrate the effectiveness of different scoring functions in ranking AFM-predicted protein–peptide complex structures. Using the Spearman correlation coefficient [53], we assessed the rank correlation of each scoring function with both other scoring functions and DockQ. To further refine the analysis, the structures predicted by AFM in both TB and TF modes were consolidated into a combined dataset (2000 models per protein–peptide complex, *i.e.*, 1000 from TB and 1000 from TF). This approach enabled the identification of a set of common candidates selected across different scoring functions, which potentially outperform other candidates in identifying near-native structures.

#### Spearman correlation between scoring functions

As shown in **Figure 6**A and B, Spearman correlation matrices were utilized to elucidate the relationships between various scoring functions in both TB and TF modes. These heatmaps illustrate that each scoring function employs a distinct strategy and algorithm to score the protein–peptide structures in our dataset. In both TB and TF modes, the internal AlphaFold-Multimer ranking score (AFM-Score) demonstrated moderate correlations with tools such as PyRosetta, FoldX-Stability, and HADDOCK. However, the correlations observed among HADDOCK variants were surprisingly modest, suggesting subtle methodological differences despite sharing a common computational framework. The minimal correlations observed between tools suggest that they are complementary and have the potential to be combined for better ranking performance. The last row in both heatmaps displays the mean correlation values of each scoring function with all other functions, excluding itself. This metric provides insight into the overall agreement and consistency of each scoring function with the others, offering a comprehensive view of their relative performance and reliability. We observed that AFM-Score exhibited the highest mean values in both TB and TF modes, followed by PyRosetta and HADDOCK scoring functions. This may indicate that the AFM-Score captures the strengths of other scoring functions in the most comprehensive way.

**Figure 6:**
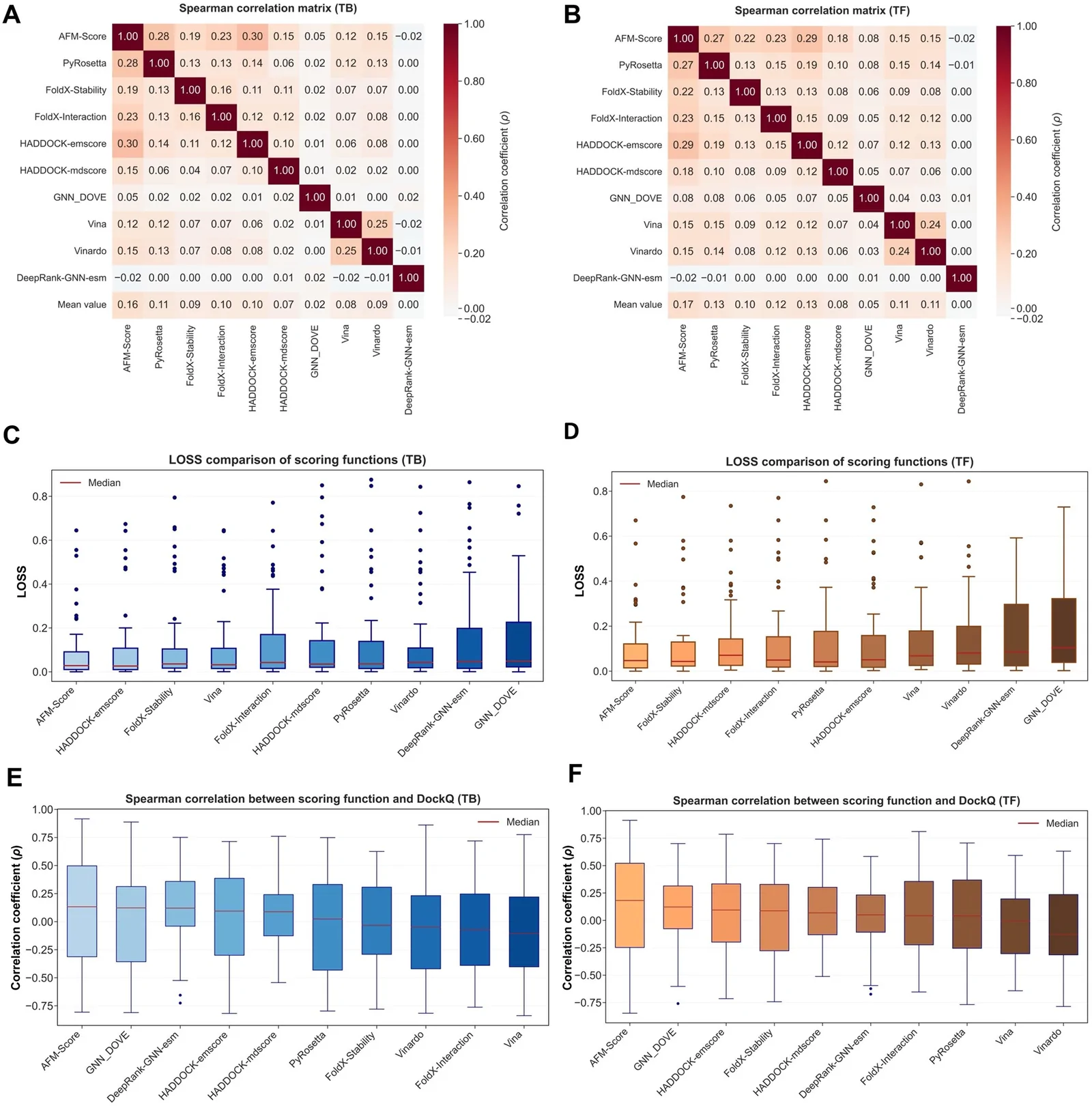
Assessment of scoring functions **A**. and **B**. Spearman correlation matrices comparing the pairwise relationships among scoring functions in TB (A) and TF (B) modes. **C**. and **D**. Box plots showing the distributions of LOSS values for protein–structure predictions by different scoring functions in TB (C) and TF (D) modes. Boxes are ordered by the mean LOSS, with the red line indicating the median. **E**. and **F**. Box plots showing the Spearman correlation coefficients between each scoring function and DockQ in TB (E) and TF (F) modes. Boxes are ordered by the mean Spearman correlation coefficient, with the red line indicating the median. AFM-Score, internal AlphaFold-Multimer ranking score.

#### Evaluation of scoring function performance via DockQ score loss

To precisely evaluate the performance of each scoring function in identifying the most near-native structure, we introduced a parameter, termed LOSS, to quantify the difference between the DockQ scores of two models: (1) the first-ranked model selected by DockQ, and (2) the first-ranked model selected by each scoring function. LOSS is defined as: LOSS = (DockQ score of the first-ranked structure by DockQ) − (DockQ score of the first-ranked structure by a scoring function).

We applied this analysis to both TB and TF modes to assess the precision of each function in ranking top-scoring structures relative to DockQ. Figure 6C shows the LOSS values of different scoring functions for TB predictions against DockQ benchmarks. AFM-Score and HADDOCK-emscore exhibited the lowest mean LOSS values of 0.087 and 0.099, respectively, indicating high accuracy and minimal variability. In contrast, DeepRank-GNN-esm and GNN_DOVE showed the highest mean LOSS values of approximately 0.160, with significant variability. PyRosetta and FoldX-Stability demonstrated intermediate performance. Figure 6D presents the LOSS values for TF predictions. DeepRank-GNN-esm and GNN_DOVE had the highest mean LOSS values of 0.173 and 0.183, respectively, suggesting lower ranking accuracy, with interquartile ranges (IQRs; representing the middle 50% of the data) of 0.273 and 0.283, respectively, reflecting significant variability. PyRosetta and FoldX-Interaction showed intermediate performance. Like in the TB case, AFM-Score had the lowest mean LOSS value of 0.101 and an IQR of 0.107. This LOSS value was higher than that in TB (0.087), probably due to the greater diversity of TF models. FoldX-Stability ranked second (mean LOSS = 0.113, IQR = 0.107), followed by HADDOCK-mdscore (mean LOSS = 0.128, IQR = 0.117). Collectively, AFM-Score outperforms other scoring functions in structure prediction and alignment with GT structures based on the DockQ score. However, it has limitations, with ten outliers in TB and/or TF modes: 7UDL, 7YUE, 7UE2, 8EBL, 8AHS, and 8C2P in both modes; 8FK3 and 7ZX4 in TB mode; and 8B58 and 8DGM in TF mode. Outliers in AFM-Score predictions may be attributed to factors such as protein complexity, data quality, model limitations, dynamics, and errors in reference structures. Identifying these outliers aids in refining predictive models.

This comprehensive analysis underscores the diversity in performance among the scoring functions, revealing that while several tools perform well, their consistency differs. The variation in the LOSS values, as indicated by the IQRs and outliers, reflects the complexity of AFM-based protein structure prediction. Nevertheless, the comparable median LOSS values of several scoring functions to that of AFM-Score suggest that multiple methods have the potential to offer similar predictive capability, though with varying levels of reliability. For example, while PyRosetta, FoldX-Stability, and HADDOCK-emscore had lower (better) median LOSS values than AFM-Score in the TF plot, AFM-Score exhibited less variability than these three functions. Detailed values for all scoring functions are listed in Tables S1 and S2.

Interestingly, AF3’s ranking of the five models may not indicate their relative performance, as shown in Figure S16. AF3 predicts many near-native structures. The number of top 10 ranking positions for AF3-predicted structures is calculated for each model as follows: first rank – 6, second rank – 12, third rank – 13, fourth rank – 8, fifth rank – 10. However, the second- and third-ranked models outperform the first-ranked model based on these numbers. This suggests that using all five AF3 models instead of just the first-ranked model may be helpful in practical applications.

#### Spearman correlation between scoring function ranking and DockQ ranking for all models

To further evaluate the overall performance of each scoring function, we analyzed the Spearman correlation coefficients [53] between each scoring function’s ranking and the DockQ ranking. In TB modeling (Figure 6E), AFM-Score led the scoring functions with a median Spearman correlation coefficient of 0.133 and a range from −0.807 to 0.915. GNN_DOVE and DeepRank-GNN-esm also exhibited strong performance, with medians of 0.123 and 0.120, respectively. In contrast, HADDOCK variants displayed more consistent but moderate performance, while PyRosetta, FoldX‑Stability, and FoldX‑Interaction showed lower and inconsistent results. High variability was observed for Vina and Vinardo. In TF modeling (Figure 6F), AFM-Score demonstrated strong adaptability, achieving a median Spearman correlation coefficient of 0.182 — higher than that in TB, probably due to a more diverse model pool in TF. GNN_DOVE ranked second, with a median of 0.123. HADDOCK variants showed moderate effectiveness and stability. DeepRank-GNN-esm demonstrated modest performance. FoldX-Interaction, Vina, and Vinardo struggled with near-zero or negative medians, highlighting the challenges in non‑template settings. Overall, robust performance is given by AFM-Score and GNN_DOVE. Detailed information about these Spearman correlation analyses is provided in Tables S3 and S4.

Additionally, the percentage of positive Spearman correlations across all scoring functions for both TB and TF modeling is illustrated in Figure S17. Although some tools showed positive correlations and higher median Spearman correlation coefficients, they did not align well with the GT DockQ ranking. For instance, despite a good overall Spearman correlation between GNN_DOVE and DockQ, GNN_DOVE’s performance in identifying the best model for each protein–peptide complex in TF modeling was weaker than that of most other tools.

In both TB and TF modeling, the highest median Spearman correlation coefficients were achieved by AFM-Score, showcasing its adaptability across diverse conditions. GNN_DOVE and DeepRank-GNN-esm displayed strong performance in TB models, but DeepRank-GNN-esm’s effectiveness was slightly reduced in analyzing the predicted structures without templates, indicating a reliance on structured data. HADDOCK demonstrated a consistent scoring approach for both TB and TF models. In less constrained environments, FoldX-Stability and FoldX-Interaction showed underperformance, highlighting their limitations. The variability of Vina and Vinardo was consistent in both contexts; however, their unpredictability was more pronounced in TF environments, emphasizing the associated risks. Overall, while some scoring functions adapt well across different frameworks, others depend significantly on the presence of templates in the prediction process, impacting their scope of application.

Because AFM-Score, FoldX-Stability, and HADDOCK-mdscore demonstrated strong performance in both TB and TF models, we selected these methods for further evaluation. In TB models, AFM-Score displayed superior overall performance with the smallest variability, whereas FoldX-Stability and HADDOCK-mdscore exhibited higher mean LOSS values and wider spreads, indicating lower robustness (Figure 6C; Table S1). In TF models, AFM-Score showed higher variability than in TB models but still maintained better performance than FoldX-Stability and HADDOCK-mdscore, which demonstrated higher mean LOSS values and wider spreads (Figure 6D; Table S2), underscoring AFM-Score’s relative superiority in TF settings despite its elevated variability relative to TB.

Our analysis reveals significant complementarity between scoring functions for evaluating protein–peptide complexes, evidenced by their weak inter-correlations. This complementarity stems from their diverse underlying algorithms: AFM-Score excels at interface quality assessment, FoldX-Stability accurately evaluates structural stability, and HADDOCK metrics reflect interaction dynamics well. The combination of FoldX-Stability with HADDOCK-mdscore proves particularly powerful, effectively compensating for each other’s limitations across diverse complexes, regardless of protein size, peptide length, or prediction difficulty. These combinations leverage the strengths of individual functions: when one function underperforms in evaluating certain structural features, another excels, resulting in more reliable structure assessment than any single function alone. Researchers can exploit these complementary relationships by strategically combining scoring functions based on their specific protein–peptide systems, thereby achieving enhanced prediction accuracy.

We combined all 1000 TB models and 1000 TF models predicted by AFM for each protein–peptide sample, and then applied each of the three scoring functions (AFM‑Score, FoldX‑Stability, and HADDOCK‑mdscore) to extract the top 10 models from each mode (*i.e.*, top 10 TB models and top 10 TF models) per sample. The Venn diagram in **Figure 7**A shows the overlap of top-ranked models among the three scoring functions, identifying common models ranked in the top 10 by all three scoring functions. These common models, characterized by consistently lower LOSS values, emerged as more reliable candidates for near-native structures than those identified by individual scoring functions. Full details of common-model values for the three scoring functions in TB and TF modes are provided in Tables S5 and S6, respectively. The intersection between FoldX‑Stability and HADDOCK‑mdscore (termed FoldX‑HADDOCK) showed the lowest mean LOSS value of 0.01 compared to all other individual or combined scoring functions (Figure 7B and C), suggesting that these two scoring functions are highly complementary for achieving a good ranking. Further analysis of the 10 common models in the FoldX-HADDOCK intersection revealed substantial diversity. These models originated from four distinct complexes (7QWV, 8DWK, 8HDJ, and 8EQ5) with protein sizes ranging from 148 to 305 residues and peptide lengths from 13 to 46 residues. The prediction difficulty varied widely across these complexes, with DockQ scores ranging from challenging (0.373–0.383 for 8DWK) and moderate (0.775–0.813 for 7QWV) to high accuracy (0.934–0.936 for 8HDJ and 0.902–0.928 for 8EQ5). Importantly, both TB and TF predictions yielded consistent low FoldX-HADDOCK LOSS values (0.0048–0.0427) across all complexes, confirming that the superior performance of this scoring combination is not biased by sample simplicity but reflects robust performance across diverse protein–peptide systems.

**Figure 7:**
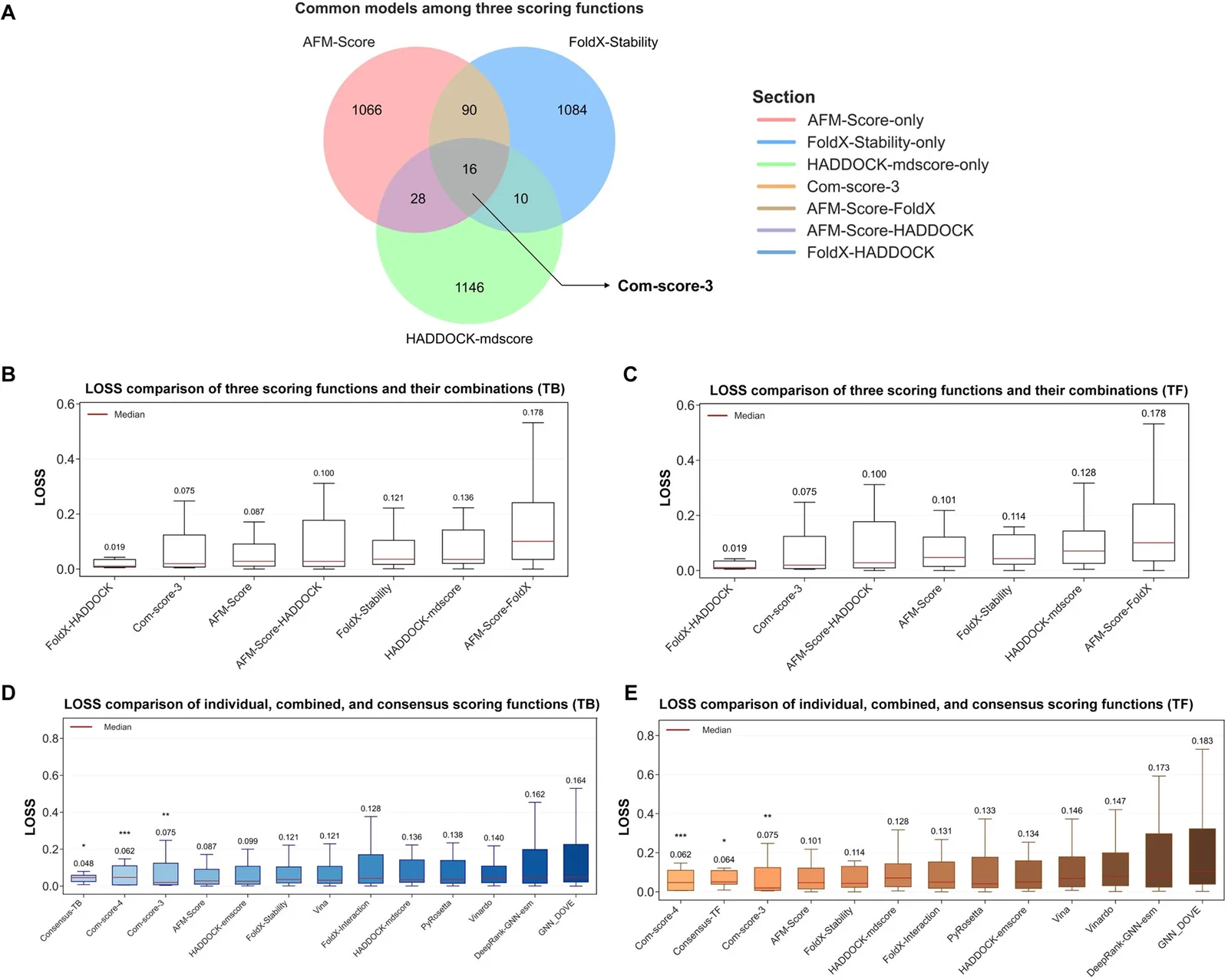
Evaluation and comparison of scoring functions for predicting near-native structures **A**. Venn diagram showing the overlap of top 10 TB and top 10 TF models for each protein–peptide complex ranked by AFM-Score, FoldX-Stability, and HADDOCK-mdscore. All scoring functions were applied to the same complete dataset of AFM-predicted structures (1000 TB and 1000 TF models per complex, 2000 in total) before top models were selected. **B**. and **C**. Box plots showing the distributions of LOSS values for protein–structure predictions by three scoring functions and their combinations (pairwise and triple) in TB (B) and TF (C) modes. Mean LOSS is labeled above each box, with a red line inside marking the median.** D**. and **E**. Box plots showing the distributions of LOSS values for protein–structure predictions by ten scoring functions, Com-score-3, Com-score-4, and the consensus method in TB (D) and TF (E) modes. Com-score-3 refers to the combination of AFM-Score, FoldX-Stability, and HADDOCK-mdscore; Com-score-4 refers to the combination of AFM-Score, FoldX-Stability, HADDOCK-mdscore, and PyRosetta. Star annotations indicate the sample size used for each evaluation: * denotes 8 samples (Consensus-TB/Consensus-TF), ** denotes 16 samples (Com-score-3), and *** denotes 6 samples (Com-score-4). Mean LOSS is labeled above each box, with a red line inside marking the median.

The combination of AFM-Score, FoldX-Stability, and HADDOCK-mdscore (termed Com-score-3) recorded a mean LOSS value of 0.07 in both TB and TF modes, lower than the mean LOSS values of the individual scoring functions: 0.08, 0.11, and 0.13 (TB) and 0.10, 0.11, and 0.12 (TF) for AFM-Score, FoldX-Stability, and HADDOCK-mdscore, respectively. As depicted in Figure 7D and E, adding PyRosetta to Com-score-3 (termed Com-score-4) achieved a mean LOSS value of 0.06, suggesting improved performance compared to Com-score-3. Both combinations outperformed all ten individual scoring functions evaluated in this study.

We also explored the potential of the consensus approach, which integrates multiple models to achieve a more reliable and accurate ranking [54]. This approach leverages the strengths and compensates for the weaknesses of individual models, leading to a more robust prediction of protein structures. For this purpose, we selected eight protein–peptide complex samples from the TB and TF datasets separately: 7WQQ, 7XTY, 8ESE, 8IA5, 8I3G, 7PRX, 8CIR, and 7R2M. These samples were obtained from different quartiles of the LOSS value distribution to ensure diverse performance representation. Each of these protein–peptide complexes has 1000 predicted structures in both TB and TF modes. We calculated the DockQ score for each of these 1000 models against all other 999 structures in the pool. Finally, we summed the DockQ scores for each structure and selected the structure with the maximum sum. This consensus approach, denoted as Consensus-TB and Consensus-TF, capitalized on the agreement among different scoring functions and exhibited superior performance (Figure 7D and E). Notably, this approach showed the smallest spread in TB models and the lowest mean LOSS values in both TB and TF models, indicating high accuracy and reliability. The analysis highlights the advantage of using a consensus approach to identify near-native structures, showing that samples highly ranked by multiple scoring functions exhibited superior performance.

## Discussion

Our work thoroughly examined AFM, CF, and AF3/AF3-L, targeting their accuracy, stereochemical quality, and reliability in predicting protein–peptide complex structures, a research area notably lacking systematic studies. Using benchmarks and reference structures from the PDB, our study provided a detailed and comprehensive evaluation of the AlphaFold family tools and their applicable scoring functions through widely adopted quality measures, including DockQ and MolProbity, across both TB and TF methodologies.

Our analysis showed that AFM excelled in TB predictions but showed moderate performance in TF precisions in the prediction pool, whereas TF outperformed TB in the first-ranked models. CF, while less accurate than AFM, was versatile and consistent in both TB and TF modes, improving slightly with templates. AF3 generated high-quality structures for more protein samples compared with both AFM and CF in the first-ranked analysis. With equal deep sampling, AF3-L consistently produced a higher proportion of high-quality structures than AFM in both TB and TF modes, shifting many predictions from medium- to high-quality categories. This advantage was particularly evident in the large-pool evaluation, highlighting AF3-L’s ability to benefit from broader conformational exploration. Our sampling-depth analysis further demonstrated that, for AFM, increasing the number of generated models in the pool substantially improved the likelihood of identifying higher-quality structures. This effect was most pronounced for complexes with initially low- or medium-quality predictions, while even high-quality starting points showed incremental gains with larger pools.

Our analysis of MolProbity scores for AFM, CF, and AF3 revealed different trends in structural prediction quality. The AFM-TB approach generally produced structures of moderate quality, and the model quality remained commendable in TF settings, even without template data. CF consistently generated medium-quality structures across both TB and TF modes, with a marginal enhancement when template data were used. The AF3 model demonstrated superior performance compared to the other two tools. In exploring specific cases like twisted peptides, AF3-predicted structures had significantly fewer twisted peptides than those from AFM or CF in first-ranked models. However, in predicting structures with *cis* non-proline peptides overall, both AFM and CF were slightly better than AF3. When evaluating all models, AF3-L also showed a much lower proportion of twisted peptides than AFM, whereas AFM was slightly better than AF3-L in limiting *cis* non-proline conformations.

It should be noted that all the evaluated structures from AF3, including those with *cis* non-proline peptides, have these conformations located on the protein part rather than on the peptide site. The analysis of hydrogen bonds in the GT and predicted structures revealed that the native structures had stronger hydrogen bonding than the predicted models. In addition, the helices generated by any model had a different distribution from those of the native structures. Despite using templates, some TB models showed more twisted peptides than the native structures and TF models. AF3 exhibited no clashes in its models, while other methods produced some clashes. Considering all factors, AF3 delivered better stereochemical quality than other methods, but still has room for improvement. Moreover, the relaxation process significantly enhanced structural quality, with Relaxed-TB and Relaxed-TF protocols reducing incorrect models while substantially lowering Clashscores. This demonstrates that the additional relaxation step in AFM effectively refines overall structural quality while maintaining prediction accuracy.

For ranking predicted models, AFM-Score is clearly better than other scoring functions. This may be partly because AFM-Score is developed explicitly for AlphaFold models, while other scoring functions are intended for general models. Some scoring functions, such as FoldX-Stability and HADDOCK-mdscore, also delivered good performance. The scoring functions are highly complementary to each other, as shown by weak correlations between any of them. This suggests the advantage of using a consensus approach to identify near-native structures. For example, common models in both three-function (Com-score-3) and four-function (Com-score-4) combinations — ranked highly by multiple scoring functions — showed better performance with lower LOSS values. Our consensus method, which extracts the structure with the highest summed DockQ score among the structures in the pool, also demonstrated better performance compared to AFM-Score and all other individual scoring functions in both TB and TF modes. Given the massive usage of AFM, it is worthwhile to design scoring functions specifically targeting structures predicted by AFM. Our study suggests the potential to refine individual scoring functions and integrate ensemble methods for improving model selection for complex structures predicted by AFM.

## Conclusion

Our study provides a comprehensive evaluation of AFM, CF, and AF3/AF3-L for predicting protein–peptide complex structures, highlighting both the strengths and limitations of each tool. AFM excels in TB predictions, while TF models often surpass TB models in terms of ranking. With equal deep sampling, AF3-L consistently produces a higher proportion of high-quality structures than AFM in both TB and TF modes. CF shows consistent performance across TB and TF modes, and AF3 demonstrates the potential for generating high-quality structures, although a deeper model pool is needed to achieve greater accuracy. Analyzing structural quality metrics such as MolProbity scores, hydrogen bonding, and specific features like twisted peptides and *cis* non-proline residues reinforces the performance differences among these tools. Notably, AF3 delivers better stereochemical quality with fewer clashes and twisted peptides, though some areas still show room for improvement. Our investigation into various scoring functions reveals the potential for optimizing structure selection through consensus approaches, combining functions such as FoldX-Stability and HADDOCK-mdscore to improve accuracy. Future efforts should focus on refining scoring functions tailored to AFM models and enhancing ensemble methods to improve prediction reliability in protein–peptide docking.

Looking ahead, several promising avenues exist for advancing protein–peptide structure prediction. These include developing specialized scoring functions optimized for protein–peptide complexes predicted by AlphaFold family tools, exploring integrative approaches that combine physics-based scoring with DL methods for improved model selection, and creating enhanced consensus strategies that leverage the complementary strengths of multiple prediction tools and scoring functions. Such developments would address current limitations while building upon established methodological strengths, ultimately improving the accuracy and applicability of protein–peptide structure prediction in structural biology and drug discovery.

## Materials and methods

### Benchmark dataset

In this study, we developed a comprehensive pipeline to evaluate the quality of protein–peptide structures predicted by AFM, CF, and AF3/AF3-L, and to assess the efficacy of various scoring functions in ranking these predicted structures and capturing protein–peptide interactions. For this purpose, we employed two quality measures together with distinct scoring functions.

To facilitate a thorough evaluation of AFM and CF’s performance in predicting protein–peptide complex structures, we initiated the systematic preparation of a benchmark dataset. We carefully curated this dataset to include protein–peptide complexes containing at least one protein and one peptide chain, with a release date after January 12, 2023 — the cutoff date for the training set used by AFM. This date threshold also ensured no overlap with AF3’s training data, as AF3 was trained on structures released on or before September 30, 2021, guaranteeing that all selected structures were novel to the predictive models. Furthermore, we restricted our selection to peptides of 3–50 residues, and excluded any sequences containing nonstandard amino acids to maintain dataset quality. Following the collection of all pertinent protein–peptide sequences, we employed the Cluster Database at High Identity with Tolerance (CD-HIT) tool [55] to remove sequences with more than 40% redundancy, thereby enhancing dataset diversity. This thorough selection process yielded a dataset of 60 unique protein–peptide complexes, ready for use in our evaluation pipeline (Table S7).

To further strengthen the assessment of model generalizability, we conducted an additional comparative analysis on our benchmark dataset, distinguishing between prediction targets with sequences similar to proteins or peptides in the training data and those without. We performed a comprehensive sequence similarity analysis and classified each complex in our benchmark as either “Unique” or “Not-Unique”. Unique structures are those without templates exceeding 30% sequence similarity in AlphaFold’s training data for protein or peptide sequences, while Not-Unique structures are those with potential homologs present in the training data within this threshold. This classification enabled us to evaluate performance stratified by homology status, providing insights into generalization capabilities for novel structures *vs.* those with template information. As shown in Figure S18, while Not-Unique structures achieved higher absolute DockQ scores, both groups exhibited similar performance trends across different confidence percentiles, with notable convergence at higher percentiles (80%–100%). This suggests that sequence similarity enhances prediction accuracy across all categories. In TF mode, which does not explicitly utilize template information, homology effects are embedded within the AlphaFold model weights.

### Model generation

AFM and AF3-L were run on NVIDIA A100 80GB PCIe GPUs, using both TB and TF modes. For each tool, default parameters were applied for multiple sequence alignment generation to maintain consistency across predictions. For each protein–peptide sample, 1000 complex structures were predicted in TB mode and another 1000 in TF mode, ensuring a comprehensive analysis. We also processed all samples using CF with its default settings under both TB and TF modes, with each mode yielding five ranked models per sample. The complex structures of all samples were also predicted using the AF3 web server, which by default returns five predicted structures per sample regardless of the presence of templates. One of the 60 samples (PDB ID: 8C2P) in our dataset could not be processed by the AF3 web server due to a biological sequence restriction, resulting in a “Sequence filtering encountered” error.

For the AFM-TB and AF3-L-TB models, the “max-template-date” parameter was set to approximately 7 days before the release date of the protein–peptide complex on the PDB website. This strategy was designed to incorporate all relevant PDB structures available in the AlphaFold dataset while explicitly excluding the target protein–peptide complex under investigation. Conversely, for TF models, the “max-template-date” parameter was adjusted to “1000-01-01”, effectively excluding all templates from the modeling process and ensuring a purely template-free approach. For CF, template use was enabled in TB mode and disabled in TF mode. Thus, AFM, AF3-L, and CF were each evaluated under both TB and TF conditions, with Amber relaxation omitted to assess only the unrelaxed, ranked models. The command-line configurations used to run AFM, AF3-L, and CF are provided below.

AFM:

Alphafold --use_gpu --data_dir=<ALPHAFOLD_DATADIR> --fasta_paths=<fasta file path> --output_dir=<output directory> --max_template_date=<max template date> --model_preset=multimer --num_multimer_predictions_per_model=200 --run_relax=false

AF3-L:

singularity exec --nv <AlphaFold3_container> python /app/alphafold3/run_alphafold.py --json_path=<input_JSON_path> --model_dir=<model_directory> --db_dir=<database_directory> --output_dir=<output_directory> --num_diffusion_samples=1000 --max_template_date=<max_template_date>

CF-TB:

colabfold_batch --templates <input_FASTA> <output_directory>

AFM models were generated using full-length sequences from the PDB SEQRES section. During preparation, we conducted a cleaning step on the predicted PDB structures to remove any residues absent from the experimental data, retaining only those present in both the modeled structures and the reference PDB ATOM section. This critical step ensured model integrity and accurate alignment with PDB sequences, which was essential for precise DockQ score calculations and reliable comparison against existing PDB entries. All structural images presented in this study were generated using PyMOL software (Schrödinger, LLC).

### Evaluation with quality measures

We used DockQ and MolProbity to compare predicted complexes to native structures and to assess their structural quality, respectively. DockQ scores, ranging from 0 to 1, assess the accuracy of predicted models against native PDB structures. According to CAPRI criteria, scores above 0.23 are considered acceptable. DockQ scores reflect the quality of the interaction interface between the protein and the peptide relative to the native structure. Figure 1 illustrates the evaluation and analysis outcomes for TB and TF modes, depicting the qualification of the top-ranked models by AFM, CF, and AF3.

For DockQ calculation, we first renumbered residues in cleaned predicted structures to match the native structure using the following command:

./DockQ/scripts/fix_numbering.pl <cleaned predicted structures> <cleaned native structure>

The output from this step was then used to compute the DockQ score as follows:

python DockQ.py <aligned predicted structures> <native structure>

DockQ scores were calculated for all predicted structures against their corresponding native structures and considered in all downstream analyses.

MolProbity is a widely used validation tool for 3D protein and nucleic acid structures. It assesses structural quality through key metrics, including Clashscores, rotamer outliers, and Ramachandran statistics, thereby ensuring model accuracy and reliability. To prepare the predicted structures for MolProbity analysis, the phenix.reduce command was executed to add hydrogens, potentially flip side chains, and build missing atoms, thus correcting common structural issues and ensuring completeness and accuracy. The processed structures were saved as <pdbfile>FH.pdb using the following command:

phenix.reduce -FLIP -BUILD <pdbfile> &> <pdbfile> FH.pdb

Subsequently, MolProbity was run on the processed structures with the command:

phenix.molprobity <pdbfile> FH.pdb

Here, <pdbfile> denotes the input PDB file.

### Evaluation with scoring functions

AFM uses an internal ranking score to rank predicted structural models, which is referred to as AFM-Score in this study. DockQ, which compares predicted models to native structures, served as a reference for evaluating the ranking accuracy of the scoring functions. All 1000 AFM-predicted models from both TB and TF modes were reranked according to their DockQ scores. To assess the ranking effectiveness of AFM-Score, we calculated the Spearman correlation coefficient between AFM’s rankings and those of DockQ. We employed a range of scoring functions to rank the AFM-predicted structures in both TB and TF modes, including PyRosetta, HADDOCK (emscore and mdscore), AutoDock Vina (Vina and Vinardo), FoldX (Stability and Interaction), GNN_DOVE, and DeepRank-GNN-esm (**Table 1**). These scoring functions were selected to analyze the predicted structures from different perspectives.

**Table 1:** Overview of scoring functions and their key features

Function	Score	Key feature	URL
HADDOCK3 [24]	emscoring, mdscoring	Flexible docking incorporating experimental data	https://github.com/haddocking/haddock3
FoldX [26]	Stability, AnalyseComplex	Perform rapid energy calculation, mutational effect prediction, protein stability analysis	https://foldxsuite.crg.eu/
PyRosetta [56]	PyRosetta	Support detailed energy manipulation and protein design	https://www.pyrosetta.org/downloads
AutoDock Vina [25]	Vina, Vinardo	Use gradient optimization techniques	https://github.com/ccsb-scripps/AutoDock-Vina
DOVE [30]	GOAP, IT-Score (ATOM40)	Integrate GOAP and IT-Score	https://github.com/kiharalab/DOVE
GNN_DOVE [31]	DOVE score	Incorporate GNN for enhanced prediction accuracy	https://github.com/kiharalab/GNN_DOVE
DeepRank [32]	Wrong Rank, Near Native	Focus on ranking PPIs using DL	https://github.com/DeepRank
DeepRank-GNN-esm [34]	DeepRank score	Combine GNN with LLMs for advanced feature learning	https://github.com/DeepRank/DeepRank-GNN-esm
InterPepRank [35]	InterPepRank score	Perform GNN-based protein prediction	https://bitbucket.org/isaakh94/interpeprank.git

*Note*: GNN, graph neural network; PPI, protein–protein interaction; DL, deep learning; LLM, large language model.

PyRosetta is a customizable toolkit for protein structure prediction and design, and we used its scoring function in this study. HADDOCK employs empirical (emscore) and molecular dynamics (mdscore) scoring for flexible docking of biomolecules. AutoDock Vina and its variant Vinardo provide efficient scoring functions based on energy calculations, considering a 5 Å grid box around the peptide ligand to ensure accurate modeling of critical interactions within the binding site while maintaining computational efficiency. GNN_DOVE and DeepRank-GNN-esm are two other DL-based scoring functions used for accurate scoring of protein complexes. The Python code for running and analyzing the predicted structures with these scoring functions is available in the GitHub repository.

FoldX offers both stability and interaction scoring functions to assess protein–peptide structures. The command pipeline used in this study is provided below.

First, the input PDB file is repaired using the following command:

foldx --command=RepairPDB --pdb=example.pdb

This command adds missing atoms, optimizes the structure, and fixes any structural issues, ensuring a complete and accurate structure for downstream analysis.

Next, the stability of the protein structure is calculated based on the repaired structure:

foldx --command=Stability --pdb=<pdbname>_Repair.pdb

This command estimates the free energy of folding, referred to as FoldX-Stability in this study, which can be used to assess the stability and reliability of the protein model.

Finally, the interactions between specific chains (in this case, chains A and B) in the repaired structure are analyzed:

foldx --command=AnalyseComplex --pdb=<pdbname>_Repair.pdb --analyseComplexChains=A,B

This command calculates multiple energy terms associated with the protein–peptide complex, such as binding energy, providing insights into the interaction dynamics and binding stability of the complex. This parameter is referred to as FoldX-Interaction in this study.

In addition to the aforementioned scoring functions, we evaluated DOVE and DeepRank [32] on all 60 samples. However, due to installation issues with DOVE, which led to unsatisfactory outcomes (predominantly zero values for ATOM40), we excluded its results from our evaluation. DeepRank, which relies on PSSM in its algorithm, encountered difficulties in identifying necessary homologs for very short peptides. As a result, it was applicable to only 28 out of the 60 samples of our dataset, leading to its exclusion from our evaluation. Other scoring functions were also attempted for installation and testing on our dataset but could not be used. GDockScore [57] was not applicable since it only accepts peptides longer than 30 residues, while some of the peptides in our dataset are shorter than 30 residues. InterEvScore [58] encountered an error when running, and alignment only works with chains longer than 30 residues as well.

We conducted a detailed assessment of computational requirements for all evaluated methods using a server equipped with Intel(R) Xeon(R) Gold 6338 CPU @ 2.00GHz and NVIDIA A100 80GB PCIe GPUs (CUDA Version 12.8). AFM required significant computational resources, with runtimes of approximately 40–60 h per sample for TB and 35–50 h for TF, utilizing ∼ 40 GB of GPU memory. In contrast, CF delivered results much faster (30–45 min for TB and 20–35 min for TF), while requiring moderate GPU resources (∼ 15 GB for TB and ∼ 12 GB for TF). AF3, being web-based, completed predictions in just 10–25 min without local hardware requirements. Storage demands also varied considerably. AFM with .pkl files required extensive storage; however, retaining only the essential PDB files reduced this to under 500 MB for all 1000 models. In contrast, CF and AF3 required less than 100 MB for their 5 models. These substantial differences in computational demands highlight important practical considerations when selecting appropriate tools based on available infrastructure and time constraints.

### Evaluation of scoring functions

Our analysis evaluated multiple scoring functions using a dataset of protein–peptide complexes, comparing AFM-predicted structures (TB and TF) against native structures for true structural fidelity. DockQ served as a reference by comparing and ranking predicted structures aligned with their native counterparts, establishing a benchmark for assessing other scoring functions. We used the Spearman correlation coefficient (*ρ*) as the metric for this evaluation. For two ranked sets of *n* observations, the coefficient is defined as:


(1) $$\rho =1-\frac{6\;\sum_{i=1}^{n}d_{i}^{2}}{n(n²-1)}$$

where $r_{i}$ is the rank of the *i*-th observation in the first set, *s_i_* is the rank of the *i*-th observation in the second set, $d_{i}=r_{i}-s_{i}$ is the difference between the ranks of corresponding values, and $\sum d_{i}^{2}$ is the sum of the squares of the rank differences [59]. We used *ρ* to determine the concordance level between the rankings produced by a range of scoring functions and the established DockQ benchmark. A *ρ* value close to 1 indicates a strong positive agreement, signifying that a scoring function’s rankings are highly consistent with those of DockQ. Such a correlation is indicative of the scoring function’s predictive reliability for the structural configurations of protein–peptide complexes.

We note that not all scoring functions provided scores for all models. AutoDock Vina failed for four samples, 8CK5, 8ESE, 8HDJ, and 8HEP, due to time and memory limitations. Consequently, AutoDock Vina evaluated a total of 56 samples. GNN_DOVE and FoldX also failed for samples 8HEP and 8ARE in both the TB and TF approaches due to time constraints encountered during model execution. As a result, these two scoring functions were evaluated on only 59 samples. InterPepRank’s inference on a CPU typically took 2–3 days per sample, indicating time inefficiency. Additional challenges included NaN values and uniform results due to incorrect weight loading, leading to prediction failures. Satisfactory results were not obtained for half of the samples in both TB and TF models. Due to this data inequality, we excluded InterPepRank from our evaluations.

### Consensus approach

This consensus approach aims to identify the predicted structure with the greatest overall similarity to all other structures in the pool and ranks it as the best prediction. For a given sample, we calculated the DockQ score for each of the 1000 predicted structures in either TB or TF mode against the remaining 999 structures in the pool. For each structure, we summed its DockQ scores against all others, and selected the one with the maximum sum. Equations 2 and 3 formalize this process:


(2) $$Sum_{i}=\;\sum_{\begin{array}{c} j=1\\ j\neq i \end{array}}^{n}\;D_{i,j}\;\;\;\;\;\;\;\mathrm{for}\;i\;\in \;\{1,\;\ldots ,\;n\}$$

(3) $$i^{*}=\text{arg}\;\underset{i\in \left\{1,\ldots ,n\right\}}{\max}\;Sum_{i}\;$$

In Equation 2, $D_{i,j}\;$represents the DockQ score between the *i*-th and *j*-th predicted structures in the pool, and i^*^ shows the maximum sum.

## Code availability

The source code of this study is freely available at https://github.com/NeginManshour/PpEv.

## Supplementary Material

qzag026_Supplementary_Data

## Data Availability

The data generated for evaluation in this study are publicly available at https://github.com/NeginManshour/PpEv.

## CRediT author statement

**Negin Manshour:** Conceptualization, Investigation, Data curation, Formal analysis, Visualization, Writing – original draft. **Jarett Zida Ren:** Investigation, Validation, Writing – review & editing. **Farzaneh Esmaili:** Investigation. **Erik Bergstrom:** Investigation. **Dong Xu:** Conceptualization, Project administration, Supervision, Resources, Funding acquisition, Writing – review & editing. All authors have read and approved the final manuscript.
